# Addition of Organic Acids and *Lactobacillus acidophilus* to the Leguminous Forage *Chamaecrista rotundifolia* Improved the Quality and Decreased Harmful Bacteria of the Silage

**DOI:** 10.3390/ani12172260

**Published:** 2022-08-31

**Authors:** Qixian Feng, Wenjiao Shi, Siqi Chen, Abraham Allan Degen, Yue Qi, Fulin Yang, Jing Zhou

**Affiliations:** 1 College of Animal Science (College of Bee Science), Fujian Agriculture and Forestry University, Fuzhou 350002, China; 2 China National Engineering Research Center of Juncao Technology, Fujian Agriculture and Forestry University, Fuzhou 350002, China; 3 Desert Animal Adaptations and Husbandry, Wyler Department of Dryland Agriculture, Blaustein Institutes for Desert Research, Ben-Gurion University of the Negev, Beer Sheva 8410500, Israel; 4 Institute of Arid Meteorology, China Meteorological Administration, Lanzhou 730020, China

**Keywords:** citric acid, *Chamaecrista rotundifolia* silage, *Lactobacillus acidophilus*, malic acid, microbial diversity

## Abstract

**Simple Summary:**

The leguminous forage *Chamaecrista rotundifolia* contains a high crude protein content and can be an important feed source for livestock. Forage is preserved mainly as silage, and microbial fermentation is key to high-quality silage. The fermentation quality of silage depends on the microbial community structure and the metabolites produced during silage fermentation. The current study provides evidence that the addition of malic or citric acid and/or *Lactobacillus acidophilus* (L) improves the quality of silage and inhibits the growth of harmful microorganisms.

**Abstract:**

This study aimed to investigate the effects of citric acid, malic acid, and *Lactobacillus acidophilus* (L) on fermentation parameters and the microbial community of leguminous *Chamaecrista rotundifolia* silage. Fresh *C. rotundifolia* was treated without any additive (CK), or with L (10^6^ CFU/g fresh weight), different levels (0.1, 0.3, 0.5, and 1% fresh weight) of organic acid (malic or citric acid), and the combinations of L and the different levels of organic acids for 30, 45, and 60 days of ensiling. The effects of malic acid and citric acid were similar during the ensiling process. Treatment with either citric or malic acid and also when combined with L inhibited crude protein degradation, lowered pH and ammonia nitrogen, and increased lactic acid concentration and dry matter content (*p* < 0.05). The neutral detergent fiber and acid detergent fiber increased initially and then decreased with fermentation time in all treatments (*p* < 0.05). Increasing the level of organic acid positively affected the chemical composition of *C. rotundifolia* silage. In addition, the addition of 1% organic acid increased the relative abundance of *Lactobacillus*, while the relative abundances of *Clostridium* and *Enterobacter* decreased at 60 days (*p* < 0.05). Moreover, both organic acids and combined additives increased (*p* < 0.05) the relative abundance of *Cyanobacteria* at 60 days of fermentation. We concluded that adding malic acid, citric acid, and L combined with an organic acid could improve the quality of *C. rotundifolia* silage and increase the relative abundance of beneficial bacteria. The addition of organic acid at a level of 1% was the most effective.

## 1. Introduction

*Chamaecrista rotundifolia*, an important leguminous forage grass, is sown for livestock fodder in tropical and subtropical regions due to its nutritive value, nitrogen fixation activity, and resistance to diseases and pests [1]. It was reported that cattle grazing native pasture plus *C. rotundifolia* gained 40% more live weight than cattle grazing native pasture alone [2]. However, fresh *C. rotundifolia* contains alkaline substances and has a high tannin content, which makes it difficult for livestock to digest and absorb. Suitable processing is required to improve its palatability, digestibility, and nutritive value.

Silage is a common way for the preservation of forages and is a crucial component of ruminant feeding. However, its safety and quality have become prerequisites for its continued development in ruminant husbandry [3]. Ensiling is a process for epiphytic lactic acid bacteria (LAB) to use water-soluble carbohydrates (WSC) in forage to produce lactic acid [4]. This process lowers the pH of the silage and inhibits the growth of undesirable microorganisms [5].

The insufficient LAB content and low WSC content of *C. rotundifolia* make it difficult to ensiling. Consequently, LAB inoculants are widely used as additives to ensure optimal fermentation [6]. For example, *Lactobacillus acidophilus* (L), a homofermentative bacteria with a pH range of 3.72–7.74 [7], has a strong inhibitory effect on food deterioration and harmful microorganisms due to the generation of lactic acid [8]. Citric acid is commonly added to silage and animal feed to improve feed utilization, and to inhibit the growth of harmful microorganisms such as *Clostridium* and *Escherichia coli* [9]. Malic acid plays a pivotal role in the succinic acid-propionic acid metabolic pathways in the rumen and possesses an antibiotic-like property that inhibits the growth of harmful microorganisms [10]. In addition, the inclusion of other electron acceptors, such as malic acid, in ruminant feed can reduce the availability of H_2_ in the rumen for methane generation without affecting rumen functions [11]. Both malic and citric acids are important intermediates of the tricarboxylic acid cycle, are involved in energy metabolism, can be used as carbon sources to supply energy for the activities of beneficial microorganisms, and can reduce the pH of silage, which creates an improved environment for silage fermentation [12]. Li et al. [13] reported that malic and citric acids could enhance the silage quality of cassava foliage, and their effectiveness improved when combined with *Lactobacillus plantarum*. The fermentation quality of silage relies, to a large extent, on the microbial community and its metabolites. Hence, further studies on the composition of microbial silage communities can provide a scientific basis for improving fermentation quality [14]. However, the impacts of malic acid, citric acid, and L on fermentation parameters and the microbial community of *C. rotundifolia* silage remain largely unknown.

This study aimed to fill this gap. In the present study, different levels of malic acid, citric acid, and L, and the organic acids in combination with L were added to *C. rotundifolia*. We hypothesized that citric acid, malic acid, and L would have beneficial effects on the fermentation and microbial community of *C. rotundifolia* silage, and there might be a potential synergistic effect when citric acid and malic acid are combined with L.

## 2. Materials and Methods

### 2.1. Silage Preparation

In September 2020, *C. rotundifolia* was harvested from Wanan Farm (Zhangpu County, Zhangzhou, China) at 8–10 cm above ground. The forage was spread out evenly indoors and dried until the dry matter (DM) reached 25.0% fresh weight (FW), which took approximately 4 h.

*C. rotundifolia* was cut into 2–3 cm lengths with a grass cutter, and treated with different additives as follows: distilled water (CK), *Lactobacillus acidophilus* (L, provided by Fujian Academy of Agricultural Sciences, Fuijan, China) at 1 × 10^6^ cfu/g fresh weight, different levels (0.1, 0.3, 0.5, and 1% FW) of malic acid (M1–M4) and citric acid (C1–C4) (analytical reagent, Shanghai Aladdin Biochemical Technology, Shanghai, China) and L combined with the different levels of malic acid (ML1–ML4) or citric acid (CL1–CL4). The additives, based on the FW of the wilted raw material, were dissolved in 10 mL of sterile water and sprayed evenly on the surface of the *C. rotundifolia* with a small spray bottle (an equal amount of distilled water was sprayed on CK). Then the *C. rotundifolia* was packed into vacuum sealed bags (24 cm × 35 cm) of 400 g each. The bags were sealed with an automatic vacuum compressor, and the *C. rotundifolia* was fermented anaerobically at a room temperature of 25 ± 3 °C. At 30, 45, and 60 days of ensiling, three bags were randomly selected from each treatment to determine nutritional components and fermentation parameters. In total, there were 9 bags (3 fermentation times × 3 replicates) for each treatment.

### 2.2. Sampling and Chemical Analysis

After 30, 45, and 60 days of fermentation, *C. rotundifolia* was removed from the bags, dried at 65 °C for 48 h for DM determination, and ground into powder to determine the chemical composition. Briefly, WSC content was measured by the anthrone-sulfuric acid colorimetric method [15]; total nitrogen (TN) content was measured by an automatic nitrogen determinator (KDN-103F, Shanghai Fiber Inspection Instrument Co., Ltd., Shanghai, China); crude protein (CP) content was measured by TN × 6.25; acid detergent fiber (ADF) and neutral detergent fiber (NDF) according to Van Soest et al. [16]; and ash content according to Monti et al. [17].

To determine fermentation parameters, 10 g samples were homogenized with 90 mL ultrapure water, sealed with sealing film, refrigerated at 4 °C for 24 h, and then filtered through four layers of medical gauze. The pH was measured by a pH meter (FiveEasy Plus, Mettler Toledo International Trading Co., Ltd., Shanghai, China). Ammonia nitrogen (NH_3_-N) content was determined by the phenol-sodium hypochlorite colorimetric method [18], and lactic, acetic, and propionic acids were determined following Wang et al. [19], using a high-performance liquid chromatograph (HPLC, WondaSil C18 Superb column, Shimadzu, Kyoto, Japan; SPD 210 nm; flow rate 1 mL/min; temperature 30 °C).

### 2.3. DNA Extraction, Amplicon Library Preparation, and Sequencing

After 60 d of fermentation, a portion of each silage sample was snap-frozen (−80 °C) for further analysis. To analyze the microbial diversity of 60 d silage, CK, L, and organic acid treatments with higher fermentation quality (C4, CL4, M4, ML4) were selected for high-throughput sequencing. Total genome DNA was extracted using the CTAB/SDS method, and DNA concentration and purity were monitored on 1 % agarose gels. The PCR was performed using diluted genomic DNA as a template, specific primers with Barcode, Phusion^®^ High-Fidelity PCR Master Mix with GC Buffer (New England Biolabs (Beijing) LTD., Beijing, China), and high performance and fidelity enzymes based on the selection of sequencing regions to ensure amplification efficiency and accuracy. The hypervariable regions V3–V4 of the bacterial 16S rDNA were obtained using 315F (CCTAYGGGRBGCASCAG) and 806R (GGACTACNNGGGTATCTAAT) as primers. The PCR products were detected by electrophoresis using 2% concentration of agarose gel, purified by magnetic beads, quantified by enzyme labeling, mixed thoroughly in equal amounts according to the concentration of PCR products, and the products were recovered for the target bands using a gel recovery kit (Qiagen, Hilden, Germany). The PCR amplification products were identified by agarose gel electrophoresis, and a MiSeq library was constructed and sequenced using TruSeq^®^ DNA PCR-Free. The library quality was assessed on the Qubit@2.0 Fluorometer (Invitrogen, Carlsbad, CA, USA) and the Agilent Bioanalyzer 2100 System (Agilent, Santa Clara, CA, USA). After the library was qualified, it was double-ended sequenced based on the Illumina NovaSeq sequencing platform to obtain a complete microbial community.

### 2.4. Sequence Processing and Analysis

The reads of each sample were spliced using FLASH after truncating the barcode and primer sequences to obtain raw tags [20]. The raw tags were processed according to the QIIME quality control process to obtain high-quality clean tags [21]. The tag sequences were compared with the species annotation database (https://github.com/torognes/vsearch/ accessed on 6 January 2021) to detect chimeric sequences, and the final effective tags were obtained by removing the chimeric sequences. Sequences were clustered into operational taxonomic units (OTUs) with 97% consistency by default for all effective tags using Uparse [22]. MUSCLE was used for a fast multiple sequence comparison to obtain the phylogenetic relationships of all OTU-representative sequences [23]. Finally, the data of each sample were homogenized, the least amount of data in the sample was used as the criterion for homogenization, and the subsequent alpha and beta diversity analyses were based on the homogenized data. QIIME (version 1.9.1) was used to calculate Observed-species, Chao1 richness estimate, Shannon diversity index, Simpson diversity index, ACE richness estimate, and Goods-coverage [24]. The R (version 2.15.3) for Windows was used to determine alpha diversity index inter-group variance, which was done separately with parametric and non-parametric tests: the T-test and Wilcox-test for two groups and the Tukey-test and Wilcox-test for more than two groups (Agricolae Package). To analyze Beta diversity, ImageGP was used for analyzing principal coordinate analysis (PCoA) based on a Bray–Curtis dissimilarity matrix [25]. Spearman’s correlation analysis tested the correlations between the main genera and silage fermentation quality.

### 2.5. Sampling and Chemical Analysis

The data were subjected to a two-way analysis of variance (SPSS software, version 26.0, Chicago, IL, USA). The fixed effects were the ensiling days, additive treatments, and the interactions between ensiling days and additive treatments. Differences among means were compared by Duncan’s multiple range test, and *p* < 0.05 was accepted as significant.

## 3. Results and Discussion

### 3.1. Effect of Additives on the Nutrient Composition of Chamaecrista Rotundifolia Silage

Low DM and WSC contents tend to cause spoilage of silage, and organic acids and LAB are often added to limit spoilage and improve silage quality. After 30 days of fermentation, the DM content of *C. rotundifolia* silage was higher with any of the additives than with CK silage (*p* < 0.05). The DM and WSC contents increased with increasing levels of organic acids; however, fermentation time had no significant effect on the DM content of the silage. The CP contents in the L, M2–M4, ML2–ML4, C2–C4, and CL2–CL4 silages were greater than in the CK silage (*p* < 0.05) at all fermentation times, with CL4 the greatest. The WSC content of M4 silage was higher (*p* < 0.05), and that of L silage was lower (*p* < 0.05) than that of CK silage. The CP and DM contents of the silages with all additives were higher than those in the CK silage. Similar changes were reported by Tao et al. [26]. Citric acid and malic acid, as intermediates in the tricarboxylic acid cycle, rapidly lower the pH, thereby inhibiting the growth and activity of harmful microbiota, which reduces the loss of DM and the degradation of CP [27]. Under anaerobic conditions, a low pH suppresses unwanted microbial activity if LAB ferments WSC into sufficient lactic acid [28], and can then preserve most of the CP in silage [29]. M4 had a greater concentration of WSC at 30 days of fermentation, and the lactic acid content was greater than that of CK, indicating that WSC content can promote the production of lactic acid during fermentation [30]. In the present study, both LAB and organic acid additions increased the lactic acid concentration and reduced the concentrations of acetic and propionic acids.

The NDF content increased initially and then decreased with time of fermentation, and after 60 days, the NDF content was lower than at 30 and 45 days (*p* < 0.05) in all treatments. The NDF content at 30 days of fermentation was lowest in the M4 silage (*p* < 0.05), and at 60 days was lowest (*p* < 0.05) in the L silage among treatments. The ADF contents of the L, M4, ML4, CL2–CL4, and C2–C4 silages were generally lower than those of CK silage (*p* < 0.05) at all fermentation times. After 60 days of fermentation, the ADF content in each treatment decreased significantly, with 1% malic acid being the lowest. At 45 days of fermentation, ML4 and CL4 treatments had lower (*p* < 0.05) ash contents than CK treatment, while at 60 days of fermentation, the ash content in all silages was lower than in CK (*p* < 0.05) (Table 1 and Table 2). The NDF and ADF contents of forages affect the digestibility of feed in ruminants. In this study, the L, M4, ML4, C4, and CL4 silages reduced the NDF and ADF contents in the silage due, at least in part, to the hydrolysis of digestible parts of cell walls by organic acids during fermentation [31]. In addition, the NDF and ADF contents in the LAB and LAB with organic acid silages were lower after 60 days than after 30 and 45 days of fermentation, which may be due to the continued hydrolysis of structural carbohydrates [32]. In general, the acid hydrolysis of structural carbohydrates is accompanied by the release of WSC, which was evident in this study [29]. The L in this study belonged to homotypic fermentative LAB, which uses more WSC to ferment into lactic acid than heterofermentative LAB. Therefore, the addition of a combination of L and citric acid or malic acid was effective in reducing fiber content and thereby improving the digestion and utilization of *C. rotundifolia* silage by herbivores. The level of 1% citric or malic acid gave the best results.

### 3.2. Effect of Additives on the Fermentation Parameters of Chamaecrista rotundifolia

Acid production and pH reduction during fermentation are spontaneous results of microbial activity and are used as important indicators in evaluating the quality of silage [33]. Grass silage pH is generally below 4.2, which is designated as excellent quality, and legume silage with high CP content has a higher pH, generally between 4.0 and 5.6 [34]. In the present study, the pH of the silage was lower (*p* < 0.05) with the addition of organic acids or LAB than the CK silage (Table 3 and Table 4) and decreased with an increase in the level of citric acid or malic acid (*p* < 0.05). The lowest pH occurred in silage with 1% citric or malic acid and with these acids plus LAB. In addition to the lowered pH, the NH_3_-N:TN ratio was below 0.1 with all additives during the 60 days of fermentation, indicating good silage quality. However, overall, fermentation time did not affect the pH or the NH_3_-N:TN ratio. The rapid reduction of NH_3_-N:TN ratios in the C4 and M4 silages may be due to the accumulation of lactic acid and the rapid acidification of silage. At 30 and 60 days of fermentation, all silages with additives had greater lactic acid concentrations than the CK silage. Similarly, Li et al. [35] reported that the addition of organic acids rapidly reduced the pH of alfalfa silage, thus inhibiting the activity of undesirable microorganisms and protease activity and resulting in lower non-protein nitrogen and NH_3_-N contents in alfalfa silage. The NH_3_-N:TN ratio decreased with increasing citric acid addition at 30 and 60 days of fermentation (*p* < 0.05), and at 45 days of fermentation, the NH_3_-N:TN ratio was lower, except for M1, in all silages than the CK silage (*p* < 0.05). The acetic acid concentration was lower in all silages than in CK, but only C3 remained lower throughout (*p* < 0.05). Citric acid and malic acid displayed similar effects in reducing acetic acid concentrations. Propionic acid concentrations in all silages were lower (*p* < 0.05) than in the CK silage at 30 and 60 ensiling days.

The addition of 0.5% and 1% citric or malic acids during 60 days of fermentation reduced the pH, NH_3_-N:TN ratios and acetic acid concentration of *C. rotundifolia* silage, increased the lactic acid content, reduced DM loss, and improved the quality of *C. rotundifolia* silage. Similarly, Ke et al. [36] reported that with the addition of *L. plantarum*, malic acid and citric acid, pH, and the NH_3_-N:TN ratio of alfalfa silage were reduced when compared to the control silage. The addition of two organic acids further improved silage quality, but *Lactobacillus plantarum* and an organic acid displayed a superimposed effect. In the present study, L and L combined with an organic acid produced small amounts of lactic acid, with no effect on days of fermentation, which is consistent with the study of Ni et al. [37]. Moreover, Muck et al. [34] reported that low levels of WSC in raw materials may reduce the improvement of silage quality by the addition of LAB. This could explain why, in the current study, silages with LAB alone or when combined with an organic acid did not produce substantial amounts of lactic acid.

### 3.3. Microbial Diversity in Chamaecrista rotundifolia Silage

**Figure 1:**
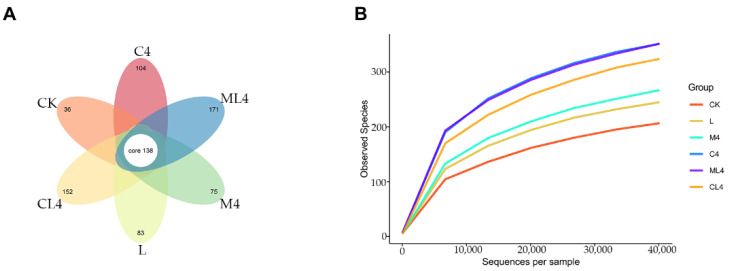
Petal diagram illustrating the degree of overlap of bacterial OTUs among the 6 groups. Each petal represents a group, the middle CORE number represents the number of OTUs common to all groups, and the number on the petal represents the number of OTUs specific to that group (**A**). Rarefaction curves of the observed species index. The horizontal coordinate is the number of sequencing strips selected randomly from a sample, and the vertical coordinate is the number of OTUs that can be constructed based on the number of sequencing strips to reflect the sequencing depth. Different samples are represented by different color curves (**B**).

The sequences were grouped in OTUs to an agreement level of 97%, and the degree of overlap of bacterial OTUs in each treatment is presented in Figure 1A. There were 1305 OTUs after 60 days of fermentation, with 138 common OTUs and 36, 83, 75, 104, 171, and 152 OTUs unique to CK, L, M4, C4, ML4, and CL4, respectively. Rarefaction curves were used to estimate species richness as a function of the sampling results. In the present study, the curves plateaued, indicating that the sequencings were saturated and that all microorganisms were identified (Figure 1B).

**Figure 2:**
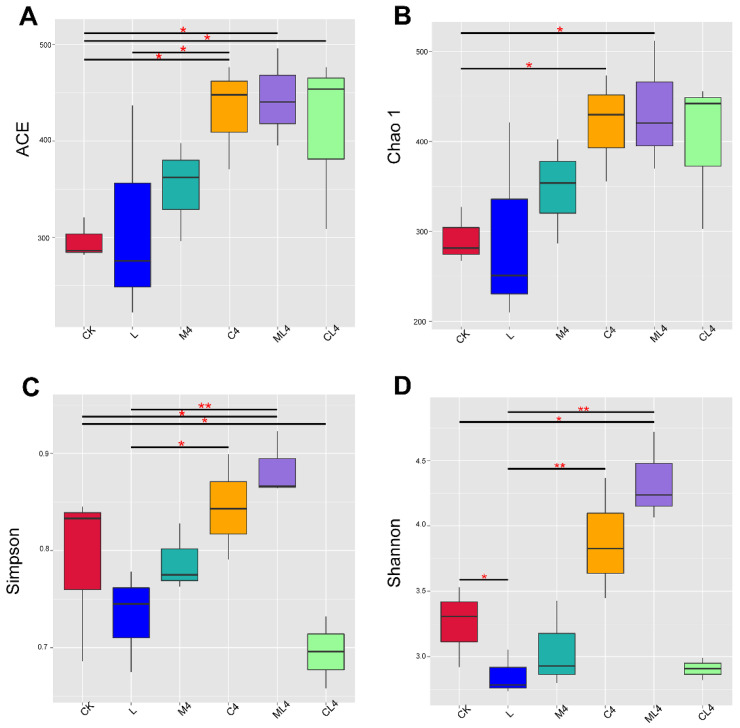
Boxplots showing the distribution of α-diversity indices among silage treatments. (**A**) ACE: Abundance Coverage-based Estimator; (**B**) Chao1 index; (**C**) Simpson’s index; and (**D**) Shannon’s diversity index. The horizontal bars in the boxes represent the median of the distance matrix distribution. The lower and upper extents of the boxes are the 25th and 75th percentiles of the distribution, respectively. The lower and upper whiskers in the box plots are the minimum and maximum values of the distribution, respectively. * *p* < 0.05; ** *p* < 0.01.

The alpha diversity of the microbial community in each silage treatment is presented in Figure 2. Chao1, an estimator of species richness based on the number of rare species, and ACE were used to estimate the number of OTUs in the community [38]. In the current study, the Shannon, Chao1, and ACE indices were lower in the L than in the CK silage, indicating that microbial diversity was reduced in the L treatment. Yang et al. [39] reported that LAB proliferated rapidly as the dominant bacteria after inoculation. In addition, a reduced pH during fermentation inhibits the activity of bacterial groups, which leads to a reduction in microbial diversity. Compared with the CK silage, the Shannon index was higher in the ML4 silage, the Chao1 and ACE indices were higher in the C4 and ML4 silages, and the ACE index was higher in the CL4 silage. This indicates that the addition of an organic acid and mixed additives could increase the bacterial community richness, which was similar to the results of Wang et al. [40]. The ACE and Chao1 indices were higher (*p* < 0.05) in M4, C4, ML4, and CL4 silages than in the CK silage, and the Chao1 index was higher (*p* < 0.05) in the C4 and ML4 silages than in the CK silage. The Shannon index was higher in the ML4 silage than the CK silage (*p* < 0.01) and was lower in the L silage than the C4 and ML4 silages (*p* < 0.05); the Simpson index was higher in the ML4 than the L silage (*p* < 0.05), and was higher in the ML4 (*p* < 0.01) and CK (*p* < 0.05) silages than the CL4 silage.

**Figure 3:**
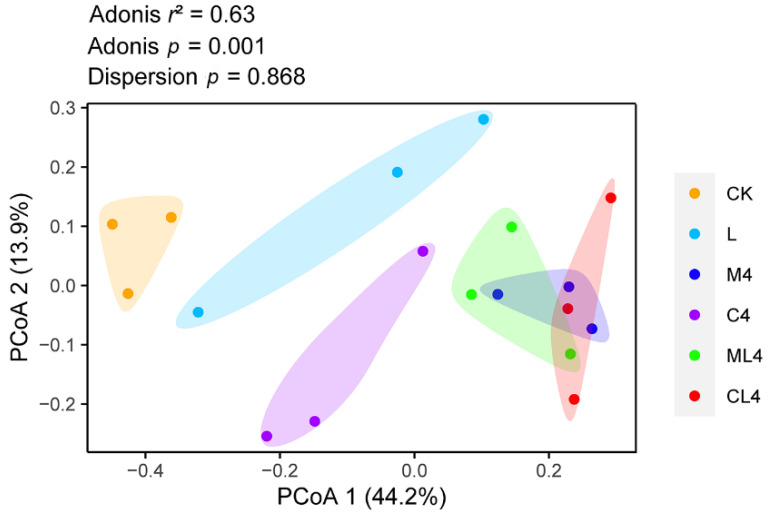
Principal Coordinates Analysis (PCoA) plot based on the Bray–Curtis dissimilarity matrix of the bacterial community among treatments. Adonis: permutational MANOVA.

Principal Coordinates Analysis (PCoA) determines and visualizes the similarities or dissimilarities of bacterial communities [41]. In this study, the PCoA plot revealed a separation and difference in bacterial communities in each ensiled treatment, indicating that the microbiota was altered with different additives (Figure 3). This difference in silage quality may be due to changes in the microbial community [42]. Therefore, based on alpha and beta diversity analyses, we concluded that malic acid, citric acid, and LAB treatments could affect the microbial diversity and community structure of *C. rotundifolia* silage.

**Figure 4:**
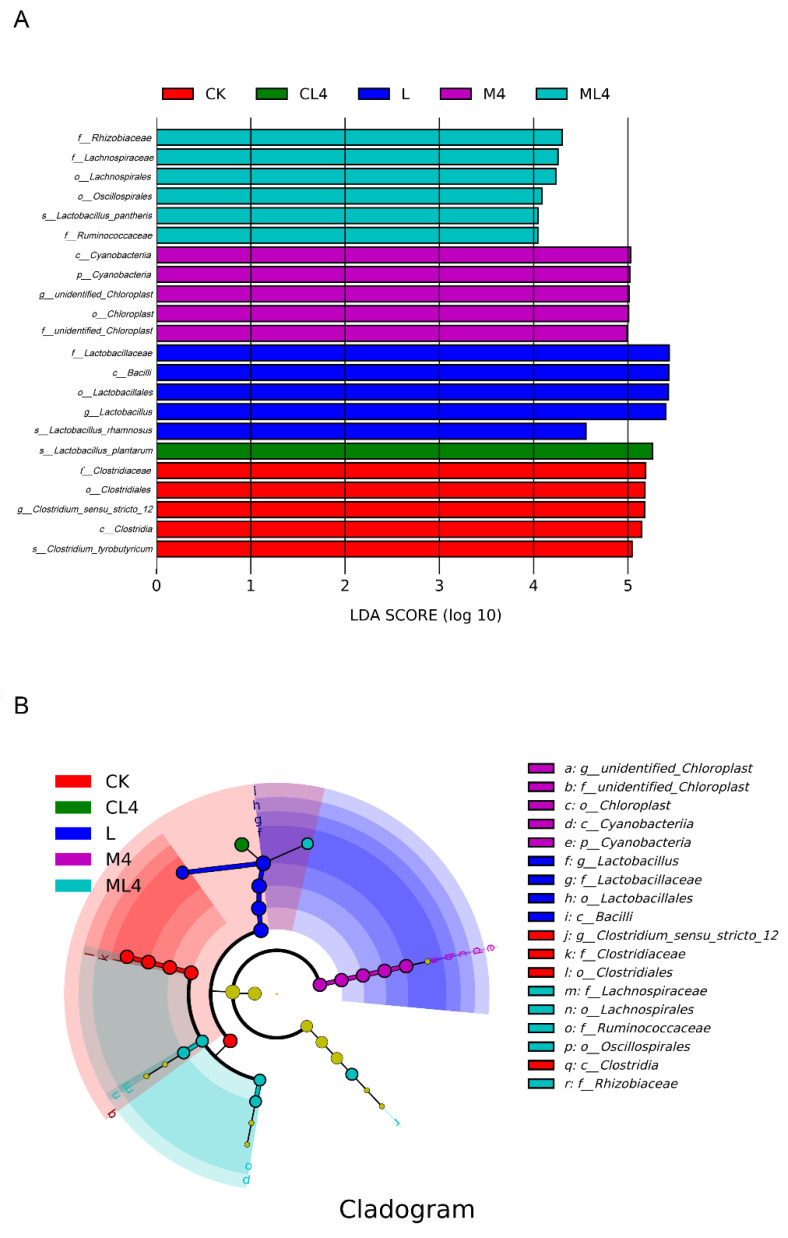
The differentially abundant bacterial taxa identified by linear discriminant analysis effect size (LEfSe) among treatments (CK, L, M4, C4, ML4, CL4) and their cladograms. (**A**) Histogram of the LDA scores. (**B**) Cladogram of LEfSe analysis. The absence of a group in the figure indicates that there are no significant different species in this group. LDA, linear discriminant analysis.

Taxonomic markers of silage *C. rotundifolia* under 6 treatments were separated based on linear discriminant effect size (LEfSe) analysis [linear discriminant analysis (LDA) score > 4] (Figure 4). There were 5 biomarkers in the CK silage, which showed *Clostridiaceae* at the family level and *Clostridium* at the genus level; 4 biomarkers in the L silage, with *Lactobacillus* at the family and genus levels; 4 biomarkers in the M4 silage, with *Cyanobacteria* at the phylum level and chloroplasts not defined at the genus level; 4 biomarkers in the M4 silage with *Cyanobacteria* at the phylum level and chloroplasts not defined at the genus level; 6 biomarkers in the ML4 silage, with *Rhizobiaceae* and *Lachnospiraceae* at the family level; one biomarker in the CL4 silage, with *Lactobacillus plantarum* at the species scale; and no biomarker in the C4 silage. Li et al. [43] analyzed differences in silage microbiome using the LEfSe method and found a significant correlation with silage fermentation.

**Figure 5:**
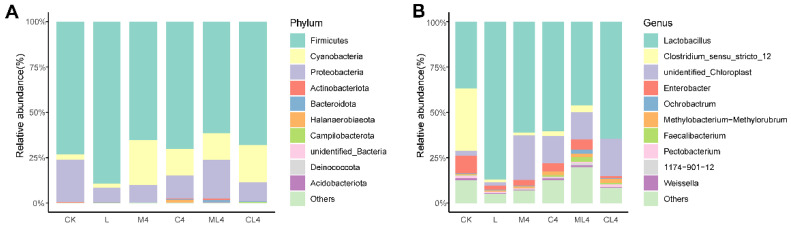
Bacterial community and relative abundances at the phylum (**A**) and genus (**B**) levels in *Chamaecrista rotundifolia* silage after 60 days of fermentation.

*Firmicutes* had the highest relative abundance of bacteria at the phylum level in CK, L, M4, C4, ML4, and CL4, with 73.2%, 89.4%, 65.4%, 70.1%, 61.4%, and 68.1%, respectively (Figure 5A). *Cyanobacteria* was a dominant phylum in M4, C4, ML4, and CL4, with 24.6%, 14.8%, 14.9%, and 20.4%, respectively, but was only 2.01% and 2.80% in L and CK silages. The highest relative abundances of the phylum *Aspergillus* in CK, M4, C4, ML4, CL4, and L silages were 23.5%, 9.70%, 12.8%, 21.1%, 10.5%, and 8.08%, respectively. In addition, ML4 had low relative abundances of *Bacteroidota* (1.13%) and *Actinobacteriota* (1.03%). Fermentation is a process of microbial colony succession. Aerobic fermentation in the pre-fermentation stage is followed by anaerobic fermentation when beneficial bacteria, mainly LAB, start to dominate. LAB produces lactic acid, which in turn inhibits the growth of undesirable colonies [44]. There are many microbial species in silage, with Firmicutes and Proteobacteria the dominant phyla, and Cyanobacteria to a lesser extent; together, they constitute the main epiphytic bacterial communities [45]. Firmicutes and Proteobacteria play an important role in the degradation of fibers in an anaerobic environment, and changes in DM, NDF, and ADF contents in silage may be related to these phyla [46]. Proteobacteria compete with LAB in the utilization of WSC, and the lower WSC content in the L, C1, C2, M1, and M2 groups may be related to this competition [47]. The relative abundance of Cyanobacteria was higher in the M4 silage than in the CK and L silages, which was attributed to the undefined chloroplasts at the genus level, presumably belonging to the plant sample fraction of *C. rotundifolia*. At the genus level, *Lactobacillus* was the dominant bacteria at 60 days of fermentation, followed by *Clostridium_sensu_stricto_12*, *Enterobacter*, *Methylobacterium-Methylorubrum*, *Faecalibacterium,* and *Pectobacterium* (Figure 5B). *Enterobacter* are non-spore forming, facultative anaerobes that are able to ferment glucose to acetic acid and other metabolites, can cause diseases in animals, such as mastitis, and can destabilize the aerobic stability of the forage [48]. In the present study, the relative abundance of *Lactobacillus* was greater in the L silage than the other silages (*p* < 0.05), and in the C4, L4, and CL4 silages was greater than in the CK silage (*p* < 0.05). The relative abundance of *Clostridium_sensu_stricto_12* was lower in all silages than the CK silage (*p* < 0.05), and of *Enterobacter* was lower in L and CL4 silages than in CK silage (*p* < 0.05). Silages with added organic acids had higher (*p* < 0.05) relative abundances of *Unidentified_Chloroplast* than CK, and ML4 silage had a higher (*p* < 0.05) relative abundance of *Faecalibacterium* than CK silage (*p* < 0.05). *Lactobacillus* spp. and *Enterococcus* spp. dominate the fermentation of feed products and improve silage quality under anaerobic conditions [49]. The dominant genus in the L, M4, C4, ML4, and CL4 silages was *Lactobacillus* spp., and the relative abundances of *Clostridium* spp. and *Enterococcus* spp. were lower than in the CK silage. This may be related to the fact that the M4, C4, ML4, and CL4 silages had increased WSC and CP contents and reduced NH_3_-N:TN ratios. *Clostridium perfringens* is detrimental to silage quality because it consumes protein and WSC. Ávila and Carvalho [50] reported that *Clostridium* spp. caused excessive protein degradation, DM loss, and butyric acid production, leading to spoilage and a reduction in silage intake by livestock. However, some *Clostridium* produce substantial amounts of butyric acid [51]. The decrease in acetic acid content in M4, C4, ML4, and CL4 may be related to the decrease in the relative abundance of *Clostridium*. The concentrations of lactic acid, acetic acid, and propionic acid and the pH may have a direct effect on bacterial activity and an indirect effect on microbial community structure [44,52]. In the present study, lactic acid and the NH_3_-N:TN ratio were correlated negatively with *Clostridium* spp. and *Enterobacter* spp.

**Figure 6:**
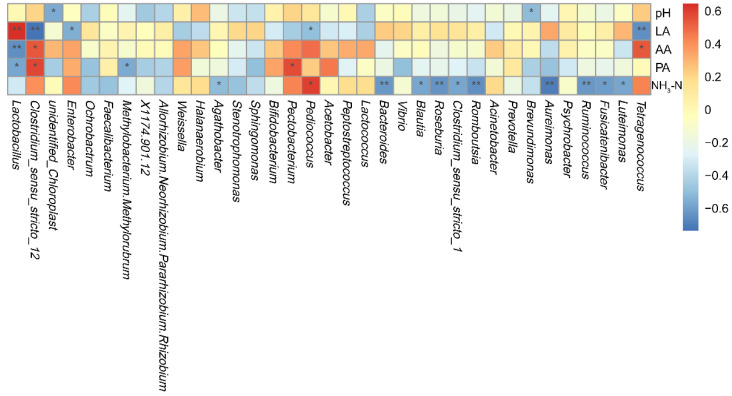
Heat map displaying the correlations between the fermentation quality of *Chamaecrista rotundifolia* silage and the relative abundance of bacterial genera. pH, hydrogen ion concentration; LA, lactic acid; AA, acetic acid; PA, propionic acid; NH_3_-N, ammonia nitrogen as a percentage of total nitrogen. * *p* < 0.05; ** *p* < 0.01.

Figure 6 presents the relationships between the ensiling fermentation quality indicators (pH, lactic acid, acetic acid, propionic acid, and NH_3_-N:TN) and microbial genera. Li et al. [14] reported that with an accumulation of lactic acid and a decrease in silage pH, *Lactobacillus* dominated, while the relative abundances of *Lactococcus*, *Enterococcus*, *Clostridium,* and *Leuconostoc* declined. In the present study. *Lactobacillus* correlated positively (*r* = 0.64) with lactic acid content, which is in agreement with Li et al. [45]. In addition, *Clostridium_sensu_stricto_12* correlated negatively with lactic acid content (*r* = −0.74) and positively with amino acids (*r* = 0.54) and propionic acid (*r* = 0.58) contents, *Enterobacter* correlated negatively with lactic acid content (*r* = −0.55), *Ruminocoocus* (*r* = -0.63), *Aureimonas* (*r* = −0.70), *Roseburia* (*r* = −0.64), and *Bacteroides* (*r* = −0.63) correlated negatively with the NH_3_-N:TN ratio, and *Pediococcus* correlated positively with the NH_3_-N:TN ratio (*r* = 0.59) and negatively with lactic acid content (*r* = −0.50).

## 4. Conclusions

When malic or citric acid was added separately or combined with L, DM loss of the silage was reduced, pH was lowered, growth, and activity of harmful bacteria were inhibited, fermentation was promoted, and protein hydrolysis was reduced. After 60 days of fermentation, Firmicutes, which enhances fiber digestion, was the dominant phylum in all treatments. The organic acids L and a combination of both increased the relative abundance of *Lactobacillus* and decreased the relative abundance of *Clostridium* and *Enterobacter*. We concluded that the addition of malic acid, citric acid, and L improved the quality of *C. rotundifolia* silage and inhibited the growth of harmful microorganisms. A level of 1% malic acid or citric acid provided the best results.

## Figures and Tables

**Table 1:** Effect of ensiling time, L, and organic acids on nutritional components of *Chamaecrista rotundifolia* silage.

Item and Ensiling Days	Treatments										SEM		*P*	
	CK	L	M1	M2	M3	M4	C1	C2	C3	C4		D	T	D × T
DM (g/kg FW)														
30	245.1 ^c^	267.3 ^a^	256.5 ^b^	262.5 ^ab^	266.4 ^ab^	271.8 ^a^	258.6 ^b^	263.1 ^ab^	268.5 ^ab^	272.1 ^a^	0.972	0.003	<0.001	1.000
45	243.6 ^c^	262.8 ^ab^	252.6 ^bc^	255.0 ^b^	260.4 ^ab^	267.6 ^a^	253.8 ^bc^	255.6 ^b^	264.0 ^ab^	267.6 ^a^				
60	246.1 ^c^	262.6 ^a^	249.9 ^bc^	257.0 ^abc^	261.3 ^ab^	266.5 ^a^	250.8 ^bc^	257.9 ^ab^	263.2 ^a^	267.4 ^a^				
CP (g/kg DM)														
30	139.0 ^Ac^	163.6 ^ab^	141.2 ^Ac^	156.7 ^Aab^	161.5 ^ab^	168.6 ^a^	152.4 ^Abc^	156.6 ^ab^	163.3 ^ab^	169.4 ^a^	1.615	<0.001	<0.001	<0.001
45	124.8 ^ABd^	159.5 ^a^	136.4 ^ABcd^	145.9 ^Bbc^	156.5 ^ab^	163.2 ^a^	138.8 ^Bc^	143.7 ^c^	157.1 ^ab^	165.1 ^a^				
60	115.1 ^Bb^	153.2 ^a^	123.2 ^Bb^	143.0 ^Ba^	152.3 ^a^	155.0 ^a^	125.2 ^Cb^	143.9 ^a^	153.4 ^a^	156.4 ^a^				
WSC (g/kg DM)														
30	11.1 ^bc^	8.2 ^c^	10.4 ^Abc^	11.3 ^bc^	11.4 ^bc^	16.9 ^Aa^	10.6 ^bc^	12.1 ^Abc^	13.0 ^ab^	13.3 ^ab^	0.339	<0.001	<0.001	0.775
45	10.3 ^bcd^	7.9 ^d^	9.4 ^ABcd^	10.4 ^bcd^	15.4 ^a^	15.1 ^ABa^	10.3 ^bcd^	12.5 ^Aabc^	13.9 ^ab^	13.0 ^abc^				
60	9.7 ^abcd^	5.0 ^f^	6.1 ^Bef^	8.0 ^cde^	9.3 ^bcd^	11.8 ^Bab^	7.2 ^def^	8.1 ^Bcde^	10.6 ^abc^	12.1 ^a^				
NDF (g/kg DM)														
30	638.1 ^Ba^	613.5 ^Abc^	646.5 ^Aa^	640.8 ^Aa^	624.3 ^Ab^	551.0 ^Bf^	620.3 ^Bb^	604.0 ^Bcd^	598.8 ^Bde^	591.7 ^Ade^	4.035	<0.001	<0.001	<0.001
45	652.5 ^Ab^	619.7 ^Ade^	671.0 ^Aa^	652.2 ^Ab^	630.5 ^Acd^	610.9 ^Aef^	639.3 ^Abc^	628.0 ^Acd^	625.5 ^Acd^	599.6 ^Ae^				
60	563.8 ^Cc^	522.6 ^Be^	604.9 ^Ba^	597.6 ^Bab^	594.9 ^Bab^	553.1 ^Bcd^	582.4 ^Cb^	549.9 ^Ccd^	546.5 ^Ccd^	542.5 ^Bd^				
ADF (g/kg DM)														
30	439.1 ^Bbc^	405.8 ^Bf^	469.4 ^Aa^	436.3 ^Bbc^	430.0 ^Bcd^	405.4 ^Bf^	448.9 ^Ab^	406.2 ^Bf^	419.7 ^Bde^	415.2 ^ABef^	2.300	<0.001	<0.001	<0.001
45	450.6 ^Ab^	435.3 ^Ac^	464.6 ^Aa^	453.8 ^Ab^	447.6 ^Ab^	414.1 ^Ad^	446.5 ^Ab^	434.7 ^Ac^	432.2 ^Ac^	425.7 ^Ac^				
60	426.6 ^Ca^	391.0 ^Bd^	435.3 ^Ba^	407.2 ^Cbc^	404.6 ^Cc^	376.0 ^Ce^	426.4 ^Ba^	413.0 ^Bbc^	414.3 ^Bb^	409.4 ^Bbc^				
Ash (g/kg DM)														
30	57.7	56.6 ^A^	52.2 ^B^	52.9 ^B^	52.9	53.7	53.7 ^B^	53.7 ^A^	55.1 ^A^	56.5 ^A^	0.444	<0.001	0.030	<0.001
45	61.2 ^a^	57.7 ^Ab^	57.8 ^Ab^	57.2 ^Ab^	53.7 ^c^	52.7 ^c^	62.7 ^Aa^	57.5 ^Ab^	57.1 ^Ab^	53.4 ^Bc^				
60	54.4 ^a^	48.4 ^Bbcd^	50.7 ^Bb^	52.5 ^Babc^	52.1 ^bcd^	52.9 ^abc^	44.8 ^Ce^	50.3 ^Bbcd^	50.9 ^Bab^	53.5 ^Bab^				

L, *Lactobacillus acidophilus*; SEM, standard error of means; DM, dry matter; FW, fresh weight; CP, crude protein; WSC, water-soluble carbohydrates; NDF, neutral detergent fibre; ADF, acid detergent fibre. *Chamaecrista rotundifolia* was treated with the following: distilled water (CK), LAB (L), and different levels (0.1, 0.3, 0.5, and 1%) of malic acid (M1–M4) and citric acid (C1–C4). D, ensilage days effect; T, treatment effect; D × T, the interaction between ensilage days and treatment. Means of additive treatment within a row (a–f) followed by different lowercase superscripts differ (*p* < 0.05). Means of ensiling time treatment within a column (A–C), followed by different uppercase superscripts, differ (*p* < 0.05).

**Table 2:** Effect of ensiling time and additives on nutritional components of *Chamaecrista rotundifolia* silage.

Item and Ensiling Days	Treatments										SEM		*p*	
	CK	L	ML1	ML2	ML3	ML4	CL1	CL2	CL3	CL4		D	T	D × T
DM (g/kg FW)														
30	245.1 ^c^	267.3 ^a^	261.6 ^b^	263.7 ^b^	269.7 ^ab^	277.8 ^a^	262.2 ^b^	265.2 ^b^	271.2 ^ab^	278.4 ^a^	1.072	0.001	<0.001	1.000
45	243.6 ^c^	262.8 ^ab^	255.0 ^bc^	257.4 ^b^	264.6 ^ab^	268.2 ^ab^	255.0 ^bc^	259.2 ^ab^	266.4 ^ab^	271.1 ^a^				
60	246.1 ^c^	262.6 ^a^	252.2 ^bc^	258.4 ^abc^	263.6 ^ab^	268.4 ^a^	252.7 ^bc^	259.4 ^ab^	264.1 ^ab^	271.2 ^a^				
CP (g/kg DM)														
30	139.0 ^Ac^	163.6 ^ab^	153.8 ^bc^	161.0 ^ab^	165.0 ^Aab^	172.8 ^Aa^	154.0 ^Abc^	161.3 ^Aab^	165.7 ^ab^	174.0 ^a^	1.659	<0.001	<0.001	0.964
45	124.8 ^ABd^	159.5 ^a^	142.4 ^d^	148.1 ^cd^	160.2 ^ABbc^	167.2 ^ABab^	142.4 ^ABd^	154.6 ^ABbcd^	165.7 ^ab^	173.7 ^a^				
60	115.1 ^Bb^	153.2 ^a^	132.5 ^c^	147.7 ^bc^	153.9 ^Bab^	158.2 ^Bab^	136.5 ^Bc^	147.7 ^Bbc^	154.1 ^ab^	168.2 ^a^				
WSC (g/kg DM)														
30	11.1 ^bc^	8.2 ^c^	9.7 ^bc^	10.9 ^abc^	11.2 ^abc^	12.7 ^a^	09.1 ^bc^	9.3 ^bc^	11.4 ^ab^	11.5 ^ab^	0.263	0.258	<0.001	0.850
45	10.3 ^bcd^	7.9 ^d^	11.2 ^ab^	11.9 ^ab^	13.0 ^a^	13.6 ^a^	10.4 ^ab^	10.9 ^ab^	11.0 ^ab^	13.4 ^a^				
60	9.7 ^abcd^	5.0 ^f^	10.0 ^a^	10.1 ^a^	12.2 ^a^	13.8 ^a^	10.3 ^a^	10.9 ^a^	12.8 ^a^	13.4 ^a^				
NDF (g/kg DM)														
30	638.1 ^Ba^	613.5 ^Abc^	608.6 ^Bcde^	602.9 ^Bcde^	597.4 ^Be^	579.8 ^Af^	629.5 ^Bab^	616.6 ^Bbc^	606.7 ^Bde^	601.1 ^Bde^	0.442	<0.001	<0.001	<0.001
45	652.5 ^Ab^	619.7 ^Ade^	657.6 ^Aa^	642.2 ^Abc^	629.5 ^Acd^	593.7 ^Ae^	641.7 ^Abc^	633.2 ^Ac^	634.9 ^Ac^	571.6 ^Ae^				
60	563.8 ^Cc^	522.6 ^Be^	577.5 ^Cc^	572.3 ^Cb^	540.5 ^Cd^	538.0 ^Bd^	595.0 ^Ca^	583.5 ^Cb^	525.8 ^Ce^	525.7 ^Ce^				
ADF (g/kg DM)														
30	439.1 ^Bbc^	405.8 ^Bf^	451.8 ^Ab^	433.1 ^Bc^	420.0 ^Bd^	415.6 ^Ade^	464.5 ^Aa^	469.0 ^Aa^	397.2 ^Cf^	404.0 ^Bef^	2.880	<0.001	<0.001	<0.001
45	450.6 ^Ab^	435.3 ^Ac^	465.5 ^Aa^	453.9 ^Aab^	449.2 ^Ab^	416.8 ^Ad^	463.6 ^Aa^	443.8 ^Bbc^	444.2 ^Abc^	416.3 ^Ad^				
60	426.6 ^Ca^	391.0 ^Bd^	394.4 ^Bc^	394.0 ^Bc^	387.3 ^Ccd^	376.5 ^Be^	416.1 ^Bb^	414.1 ^Cb^	411.1 ^Bb^	383.2 ^Cde^				
Ash (g/kg DM)														
30	57.7	56.6 ^A^	54.4	58.8 ^A^	58.1	59.7 ^A^	57.8 ^AB^	58.3 ^A^	60.0 ^A^	61.6 ^A^	0.476	<0.001	0.104	<0.001
45	61.2 ^a^	57.7 ^Ab^	54.0 ^bc^	54.1 ^ABbc^	54.1 ^bc^	49.7 ^Bc^	60.1 ^Aa^	53.7 ^Bbc^	51.8 ^Cc^	49.8 ^Cc^				
60	54.4 ^a^	48.4 ^Bbcd^	52.6 ^d^	52.6 ^Bd^	54.6 ^bcd^	55.6 ^ABbc^	53.7 ^Bbcd^	53.1 ^Bcd^	56.2 ^Bab^	58.4 ^Ba^				

SEM, standard error of means; DM, dry matter; FW, fresh weight; CP, crude protein; WSC, water-soluble carbohydrates; NDF, neutral detergent fibre; ADF, acid detergent fibre. *Chamaecrista rotundifolia* was treated with the following: distilled water (CK), LAB (L), and different levels (0.1, 0.3, 0.5, and 1%) of malic acid (ML1–ML4) and citric acid (CL1–CL4). D, ensilage days effect; T, treatment effect; D × T, the interaction between ensilage days and treatment. Means of additive treatment within a row (a–f) followed by different lowercase superscripts differ (*p* < 0.05). Means of ensiling time treatment within a column (A–C), followed by different uppercase superscripts, differ (*p* < 0.05).

**Table 3:** Effect of ensiling time, and *Lactobacillus acidophilus* and organic acids on fermentation characteristics of the *Chamaecrista rotundifolia* silages.

Item and Ensiling Days	Treatments										SEM		*p*	
	CK	L	M1	M2	M3	M4	C1	C2	C3	C4		D	T	D × T
Lactic acid (% DM)														
30	0.52 ^f^	1.44 ^bc^	1.53 ^Aabc^	0.99 ^de^	1.70 ^Aab^	1.26 ^Acd^	0.77 ^ef^	1.72 ^ab^	1.50 ^abc^	1.85 ^Aa^	0.048	0.006	<0.001	0.017
45	0.45 ^c^	1.30 ^ab^	0.89 ^Cbc^	1.37 ^ab^	1.29 ^Bab^	1.73 ^ABa^	0.95 ^bc^	1.18 ^ab^	1.21 ^ab^	1.21 ^Bab^				
60	0.44 ^c^	1.50 ^ab^	1.28 ^Bb^	1.63 ^ab^	1.56 ^ABab^	2.01 ^Ba^	1.26 ^bb^	1.51 ^ab^	1.41 ^b^	1.37 ^ABb^				
Acetic acid (% DM)														
30	1.83 ^a^	1.37 ^ab^	1.69 ^ab^	1.55 ^ab^	1.30 ^Aabc^	1.24 ^Abc^	1.78 ^ab^	1.54 ^ab^	0.82 ^Ac^	1.65 ^Aab^	0.057	<0.001	<0.001	0.334
45	1.63 ^a^	0.92 ^bc^	1.58 ^a^	1.47 ^a^	1.38 ^Aab^	1.50 ^Aa^	1.47 ^a^	1.41 ^ab^	0.54 ^Ac^	1.34 ^Aab^				
60	1.91 ^a^	1.09 ^ab^	1.44 ^ab^	0.92 ^ab^	0.41 ^Bb^	0.49 ^Bb^	1.52 ^ab^	1.02 ^ab^	0.59 ^ABb^	0.61 ^Bb^				
Propionic acid (% DM)														
30	0.24 ^Ba^	0.11 ^ABc^	0.18 ^b^	0.07 ^Acde^	0.06 ^Bde^	0.09 ^cd^	0.09 ^ABcd^	0.10 ^Bcd^	0.04 ^e^	0.04 ^Be^	0.001	<0.001	<0.001	,0.001
45	0.16 ^Ca^	0.05 ^Bb^	0.13 ^a^	0.04 ^ABb^	0.04 ^ABb^	0.02 ^b^	0.03 ^Bb^	0.15 ^Aa^	0.03 ^b^	0.04 ^Bb^				
60	0.38 ^Aa^	0.17 ^Ab^	0.13 ^bcd^	0.02 ^Bd^	0.02 ^Bd^	0.04 ^cd^	0.15 ^Abc^	0.19 ^Ab^	0.03 ^cd^	0.22 ^Ab^				
pH														
30	5.16 ^a^	4.50 ^c^	4.84 ^b^	4.63 ^c^	4.53 ^c^	4.36 ^ABd^	4.89 ^b^	4.61 ^c^	4.51 ^c^	4.35 ^ABd^	0.028	<0.001	<0.001	0.988
45	5.25 ^a^	4.54 ^cde^	4.87 ^b^	4.66 ^cd^	4.50 ^def^	4.38 ^Afg^	4.84 ^b^	4.68 ^c^	4.51 ^def^	4.37 ^Ag^				
60	5.15 ^a^	4.45 ^c^	4.70 ^b^	4.57 ^c^	4.44 ^c^	4.24 ^Bd^	4.75 ^b^	4.56 ^c^	4.47 ^c^	4.29 ^Bd^				
NH_3_-N:TN														
30	4.96 ^Aab^	4.86 ^Ab^	5.07 ^Aa^	2.82 ^Ae^	3.00 ^Ad^	1.80 ^Ah^	3.57 ^Ac^	3.05 ^Ad^	2.42 ^Af^	2.07 ^Ag^	0.135	<0.001	<0.001	<0.001
45	6.16 ^Bb^	5.32 ^Bc^	6.63 ^Ba^	4.22 ^Bd^	4.00 ^Be^	3.31 ^Bg^	3.58 ^Af^	4.07 ^Bde^	3.13 ^Bh^	2.38 ^ABi^				
60	6.20 ^Ba^	4.58 ^Abc^	5.83 ^Ca^	3.96 ^Bcd^	4.47 ^Bbc^	2.97 ^Cef^	5.01 ^Bb^	4.17 ^Bc^	3.38 ^Bde^	2.60 ^Bf^				

L, *Lactobacillus acidophilus*; SEM, standard error of means; NH_3_-N:TN, ammoniacal nitrogen as a percentage of total nitrogen. *Chamaecrista rotundifolia* was treated with the following: distilled water (CK), L, and different levels (0.1, 0.3, 0.5, and 1%) of malic acid (M1–M4) and citric acid (C1–C4). D, ensilage days effect; T, treatment effect; D × T, the interaction between ensilage days and treatment. Means of additive treatment within a row (a–i) followed by different lowercase superscripts differ (*p* < 0.05). Means of ensiling time treatment within a column (A–C), followed by different uppercase superscripts, differ (*p* < 0.05).

**Table 4:** Effect of ensiling time and additives on fermentation characteristics of *Chamaecrista rotundifolia* silage.

Item and Ensiling Days	Treatments										SEM		*p*	
	CK	L	ML1	ML2	ML3	ML4	CL1	CL2	CL3	CL4		D	T	D × T
Lactic acid (% DM)														
30	0.52 ^d^	1.44 ^c^	1.57 ^Abc^	1.62 ^bc^	1.61 ^bc^	2.66 ^a^	2.08 ^Aabc^	2.19 ^Aab^	1.99 ^bc^	1.97 ^Abc^	0.060	0.003	<0.001	0.199
45	0.45 ^d^	1.30 ^bc^	0.93 ^Bcd^	1.56 ^ab^	1.65 ^ab^	1.91 ^a^	1.25 ^Bbc^	1.38 ^Babc^	1.66 ^ab^	1.45 ^Babc^				
60	0.44 ^b^	1.50 ^a^	1.69 ^Aa^	1.99 ^a^	1.80 ^a^	1.97 ^a^	1.34 ^ABa^	1.65 ^ABa^	1.69 ^a^	1.76 ^ABa^				
Acetic acid (% DM)														
30	1.83 ^a^	1.37 ^b^	1.46 ^ab^	1.35 ^b^	0.65 ^Bd^	0.84 ^Bd^	1.28 ^bc^	1.16 ^Abc^	0.54 ^d^	0.82 ^Bcd^	0.051	<0.001	<0.001	<0.001
45	1.63 ^a^	0.92 ^c^	1.48 ^ab^	1.26 ^abc^	1.16 ^Abc^	1.21 ^Aabc^	1.32 ^abc^	1.19 ^Abc^	0.49 ^d^	1.17 ^Abc^				
60	1.91 ^a^	1.09 ^bc^	0.92 ^bcd^	0.83 ^bcd^	0.35 ^Bcd^	0.31 ^Cd^	1.33 ^ab^	0.83 ^Bbcd^	0.45 ^cd^	0.48 ^Bcd^				
Propionic acid (% DM)														
30	0.24 ^Ba^	0.11 ^ABb^	0.05 ^Bc^	0.05 ^ABc^	0.04 ^c^	0.05 ^Bc^	0.04 ^c^	0.04 ^c^	0.03 ^Bc^	0.09 ^ABb^	0.009	<0.001	<0.001	<0.001
45	0.16 ^Ca^	0.05 ^Bbc^	0.04 ^Bbc^	0.03 ^Bbc^	0.02 ^c^	0.02 ^Cc^	0.03 ^bc^	0.04 ^bc^	0.06 ^Ab^	0.06 ^Bbc^				
60	0.38 ^Aa^	0.17 ^Ab^	0.12 ^Abcd^	0.05 ^Ade^	0.03 ^e^	0.18 ^Ab^	0.08 ^cde^	0.02 ^e^	0.04 ^ABe^	0.14 ^Abc^				
pH														
30	5.16 ^a^	4.50 ^c^	4.73 ^ABb^	4.52 ^c^	4.42 ^ABc^	4.26 ^ABd^	4.66 ^b^	4.48 ^c^	4.45 ^c^	4.28 ^ABd^	0.028	<0.001	<0.001	0.999
45	5.25 ^a^	4.54 ^c^	4.76 ^Ab^	4.55 ^c^	4.49 ^Ac^	4.34 ^Ad^	4.70 ^b^	4.54 ^c^	4.50 ^c^	4.35 ^Ad^				
60	5.15 ^a^	4.45 ^bc^	4.58 ^Bb^	4.47 ^bc^	4.33 ^Bcd^	4.19 ^Bd^	4.56 ^b^	4.40 ^c^	4.39 ^c^	4.22 ^Bd^				
NH_3_-N:TN														
30	4.96 ^Aa^	4.86 ^Aa^	3.92 ^Ab^	3.31 ^d^	2.24 ^Ae^	2.13 ^e^	3.64 ^Ac^	3.38 ^cd^	3.19 ^d^	1.97 ^Ae^	0.131	<0.001	<0.001	<0.001
45	6.16 ^Ba^	5.32 ^Bc^	5.84 ^Bb^	3.34 ^e^	2.97 ^Bf^	2.41 ^g^	4.57 ^Bd^	3.37 ^e^	3.31 ^e^	2.06 ^ABh^				
60	6.20 ^Ba^	4.58 ^Ab^	4.66 ^Cb^	3.57 ^c^	2.76 ^Cde^	2.16 ^e^	4.44 ^Bb^	3.64 ^c^	3.04 ^cd^	2.26 ^Be^				

SEM, standard error of means; NH_3_-N:TN, ammoniacal nitrogen as a percentage of total nitrogen. *Chamaecrista rotundifolia* was treated with the following: distilled water (CK), *Lactobacillus acidophilus* (L), different levels (0.1, 0.3, 0.5, and 1%) of malic acid (ML1–ML4) and citric acid (CL1–CL4). D, ensilage days effect; T, treatment effect; D × T, the interaction between ensilage days and treatment. Means of additive treatment within a row (a–h) followed by different lowercase superscripts differ (*p* < 0.05). Means of ensiling time treatment within a column (A–C), followed by different uppercase superscripts, differ (*p* < 0.05).

## Data Availability

The data presented in this study are available on request from the corresponding author.
